# Zika Virus Hijacks Extracellular Vesicle Tetraspanin Pathways for Cell-to-Cell Transmission

**DOI:** 10.1128/mSphere.00192-21

**Published:** 2021-06-30

**Authors:** Sara B. York, Li Sun, Allaura S. Cone, Leanne C. Duke, Mujeeb R. Cheerathodi, David G. Meckes

**Affiliations:** a Florida State University College of Medicine, Department of Biomedical Sciences, Tallahassee, Florida, USA; University of Arizona

**Keywords:** Zika virus, extracellular vesicles, flavivirus, exosomes, tetraspanins, CD63, Zika, microvesicles

## Abstract

Extracellular vesicles (EVs) are membrane-encapsulated structures released by cells which carry signaling factors, proteins, and microRNAs that mediate intercellular communication. Accumulating evidence supports an important role of EVs in the progression of neurological conditions and both the spread and pathogenesis of infectious diseases. It has recently been demonstrated that EVs from hepatitis C virus (HCV)-infected individuals and cells contained replicative-competent viral RNA that was capable of infecting hepatocytes. Being a member of the same viral family, it is likely the Zika virus also hijacks EV pathways to package viral components and secrete vesicles that are infectious and potentially less immunogenic. As EVs have been shown to cross blood-brain and placental barriers, it is possible that Zika virus could usurp normal EV biology to gain access to the brain or developing fetus. Here, we demonstrate that Zika virus-infected cells secrete distinct EV subpopulations with specific viral protein profiles and infectious genomes. Zika virus infection resulted in the enhanced production of EVs with various sizes and densities compared to those released from noninfected cells. We also show that the EV-enriched tetraspanin CD63 regulates the release of EVs and Zika viral genomes and capsids following infection. Overall, these findings provide evidence for an alternative means of Zika virus transmission and demonstrate the role of EV biogenesis and trafficking proteins in the modulation of Zika virus infection and virion morphogenesis.

**IMPORTANCE** Zika virus is a reemerging infectious disease that spread rapidly across the Caribbean and South America. Infection of pregnant women during the first trimester has been linked to microcephaly, a neurological condition where babies are born with smaller heads due to abnormal brain development. Babies born with microcephaly can develop convulsions and suffer disabilities as they age. Despite the significance of Zika virus, little is known about how the virus infects the fetus or causes disease. Extracellular vesicles (EVs) are membrane-encapsulated structures released by cells that are present in all biological fluids. EVs carry signaling factors, proteins, and microRNAs that mediate intercellular communication. EVs have been shown to be a means by which some viruses can alter cellular environments and cross previously unpassable cellular barriers. Thus, gaining a greater understanding of how Zika virus affects EV cargo may aid in the development of better diagnostics, targeted therapeutics, and/or prophylactic treatments.

## INTRODUCTION

Zika virus (ZIKV) is a single-stranded, positive-sense RNA virus (ss+RNA) belonging to the *Flaviviridae* family (1). This reemerging arbovirus is primarily transmitted to humans by *Aedes* species mosquitos, but additional evidence indicates that it can be transmitted sexually and vertically from mother to fetus (2, 3). Historically, ZIKV has been classified as a self-limiting febrile disease, and until more recently, reported human cases have been sporadic and clustered in specific geographical regions. Since the 2015 outbreak in Brazil, the virus has continued to spread in the Western Hemisphere and the number of Zika virus infections surged with reports of neurological diseases, such as Guillain-Barré syndrome and congenital microcephaly associated with infection (4–6). The increased global threat of Zika congenital syndrome and Zika-associated neuropathies prompted the scientific community to gain a greater understanding of Zika virus replication and pathogenesis in the host. However, the important question of how this flavivirus is able to cross placental and blood-brain barriers (BBBs) to infect neural progenitor cells of the developing fetus remains unanswered.

Extracellular vesicles (EVs) are a heterogeneous mixture of communicative vehicles secreted by cells in both healthy and pathogenic circumstances. EVs are typically classified based on size as well as cellular origin and include apoptotic bodies, microvesicles, and exosomes. Apoptotic bodies are large vesicles shed by cells undergoing apoptosis whereas microvesicles are considered to be between 100 and 1,000 nm in size and formed by budding at the cell surface (7, 8). Exosomes range between 40 and 150 nm in size and are derived endocytically through budding events inside the multivesicular body (MVB) (8). In addition to these major classes of vesicles, various microvesicle and exosome subpopulations likely exist with distinct cargos and functions (9).

Many viruses, including some flaviviruses, have been found to hijack EV biogenesis pathways for virus assembly and egress or the secretion of specific viral RNAs and proteins (10–14). More recently, EVs have been found to be a unique means of transmission of certain enveloped and even nonenveloped viruses distinct from virion-mediated infection (15–17). There is a striking similarity between noninfectious EVs and virions. EVs from noninfected cells also resemble EVs from infected cells and can contain viral proteins and genome fragments (18). Historically, virologists have referred to these EVs as noninfectious or defective particles. The similar biophysical properties make true separation of virions from EVs very difficult. In this study, we were interested in analyzing EV subpopulations for Zika viral proteins and RNA to determine the effect of infection on all EV populations.

EVs arise from every cell type, are present in all bodily fluids (19, 20), and have even been found to cross the blood-brain barrier (21, 22) and the placenta (23, 24). Pregnancy increases the circulation of fetal EVs in maternal blood as early as the first trimester (23), which also coincides with the highest reported risk of infecting the fetus with ZIKV (25). Since virally modified EVs have been found to manipulate cellular microenvironments and enhance virus transmission and pathogenicity (26–28), it is conceivable that EVs released from Zika virus-infected cells may similarly affect the host.

How exactly viruses influence the cargo and functions of EVs is largely unknown. Nonetheless, it is likely at least in part by interactions between viral proteins and host EV trafficking and biogenesis pathways. Multiple pathways have been described in the literature. For instance, the endosomal sorting complex required for transport (ESCRT) pathway is important for invagination of the MVB to form the intraluminal vesicles, which become exosomes when released from the cell (29, 30). However, exosome biogenesis and release can also occur in an ESCRT-independent manner, as tetraspanins and the lipid ceramide have been shown to be important in exosome biogenesis and protein trafficking to the MVB (31, 32). Tetraspanins are a group of proteins containing four transmembrane domains, two extracellular loops, large and small, and three short intracellular regions (33). Tetraspanins are essential for many different cellular mechanisms, such as immune function (34) or trafficking of partner proteins to different organelles (35). Both ESCRT proteins and tetraspanin proteins are enriched in exosomes (36, 37).

There have been multiple established associations between viruses and EV biogenesis pathways. Human immunodeficiency virus (HIV), herpes simplex virus 1 (HSV-1), Ebola virus, and rabies virus have been previously reported to hijack parts of the ESCRT pathway for assembly and egress (38–40). Additionally, human herpesvirus 6 (HHV-6) is believed to use the tetraspanin CD63 during replication (41). Other flaviviruses, such as dengue virus, were found to recruit several host ESCRT subunits to sites of replication, and it was found that these ESCRT subunits are required for efficient viral budding (42). Here, we sought to determine if Zika virus utilizes EV biogenesis pathways for virus replication and spread. Our overall hypothesis is that ZIKV usurps EV trafficking pathways in order to package viral genomic RNA and proteins to increase infectivity, evade the host immune response, and possibly alter tropism of the virus.

## RESULTS

### Zika virus specifically modifies small EV density, cargo, and secretion.

**FIG 1:**
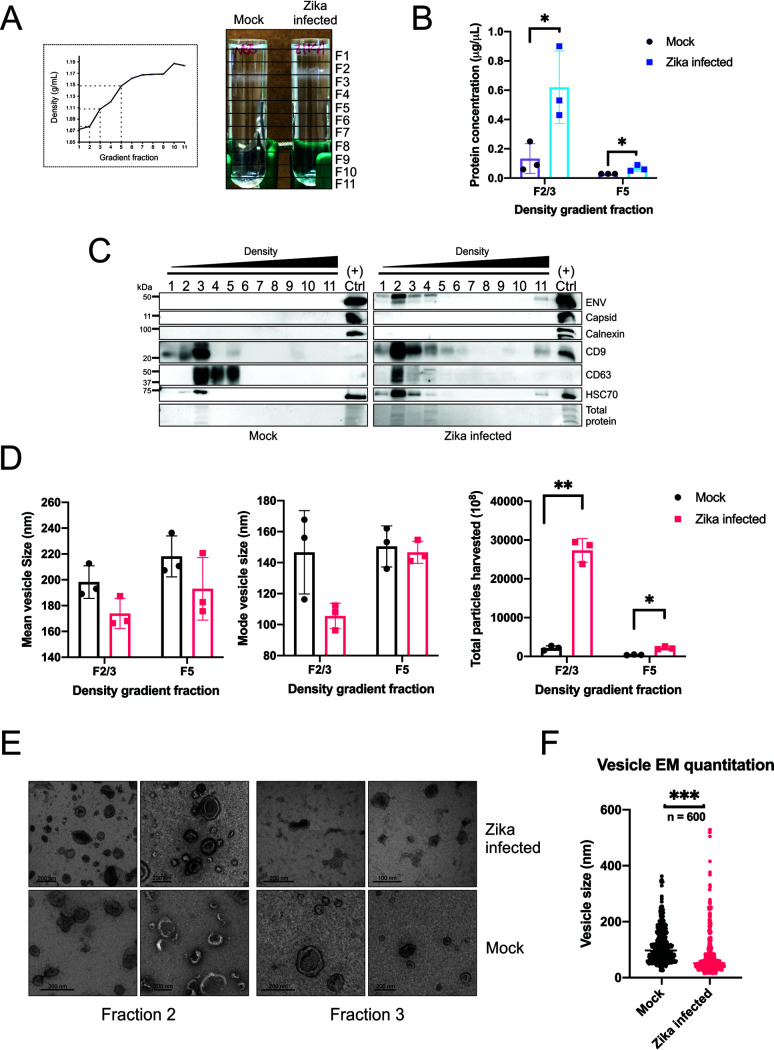
Zika virus specifically modifies small EV density, cargo, and secretion. EV-depleted medium (50 to 100 ml) harvested from SNB-19 glioblastoma (36e6 to 72e6) cells infected with Zika virus (MOI = 0.05 to 0.1) or mock infected for 72 h underwent differential centrifugation and PEG precipitation. The 100K EV pellet was further purified in a density gradient. (A) Densities of the fractions were measured with a refractometer, and visual differences between the uninfected and Zika virus-infected gradients were captured. (B) Protein quantitation of fractions 2/3 and 5 from Zika virus-infected and uninfected cells (*, *P* < 0.05). (C) Equal volumes of the uninfected and Zika virus-infected density fractions were separated by SDS-PAGE and immunoblotted for Zika virus proteins, tetraspanins, and EV markers. Positive control is the total vesicles from Zika virus-infected cells following 1,000 × *g* spin for 10 min and PEG precipitation. (D) The size and numbers of vesicles from combined fractions 2/3 and 5 were further examined by nanoparticle tracking analysis (NTA) (*, *P* < 0.05; **, *P* < 0.01; ***, *P* < 0.001). (E) Electron microscopy representative images of the vesicle-containing fractions from uninfected versus Zika virus-infected cells. (F) Quantification of vesicle sizes of the density fraction 2/3 EVs from the EM images (*n* = 600; *P* < 0.001). All error bars depict standard deviations from at least three independent experiments.

In order to investigate whether Zika virus alters small EV populations, we isolated EVs by differential centrifugation with polyethylene glycol (PEG) precipitation and further purified the small EVs with a slightly modified iodixanol gradient method originally described by Kowal and colleagues (9, 43). For the gradient, EVs are loaded on the bottom of ultracentrifuge tubes and allowed to migrate up (i.e., float) into the gradient to their appropriate densities (9, 43). The paper by Kowal and colleagues reported that small EVs travel to fractions 3 and 5, corresponding to approximately 1.11 and 1.15 g/ml, respectively (9, 43). The EVs in fraction 3, termed small light EVs, were found to represent an enrichment in bona fide exosomes of endosomal origin. The densities of the fractions from our gradient were measured and confirmed to yield similar densities as those previously described in the work of Kowal et al. (9) (Fig. 1A).

Visual differences in the EV band size and location were immediately evident (Fig. 1A). Protein quantitation of the combined fractions 2 and 3 and fraction 5 demonstrated that the total protein level is significantly higher in the fraction EVs from the Zika virus-infected cells, corroborating the presence of more EV material (Fig. 1B). Immunoblotting confirmed that EV markers and Zika virus envelope were primarily present in fraction 2 as opposed to fraction 3 in the mock infection (Fig. 1C). The gradient purification also revealed that Zika virus capsid protein failed to be detected in the fractions (Fig. 1C). The nanoparticle tracking analysis (NTA) and electron microscopy (EM) images showed that Zika virus increases the secretion of significantly smaller EVs (Fig. 1D to F). Altogether, these data indicate that Zika virus is varying the secretion and cargo of small light EVs consistent with the features and markers of exosomes (9).

### Zika virus-modified small EVs contain viral RNA and are infectious.

**FIG 2:**
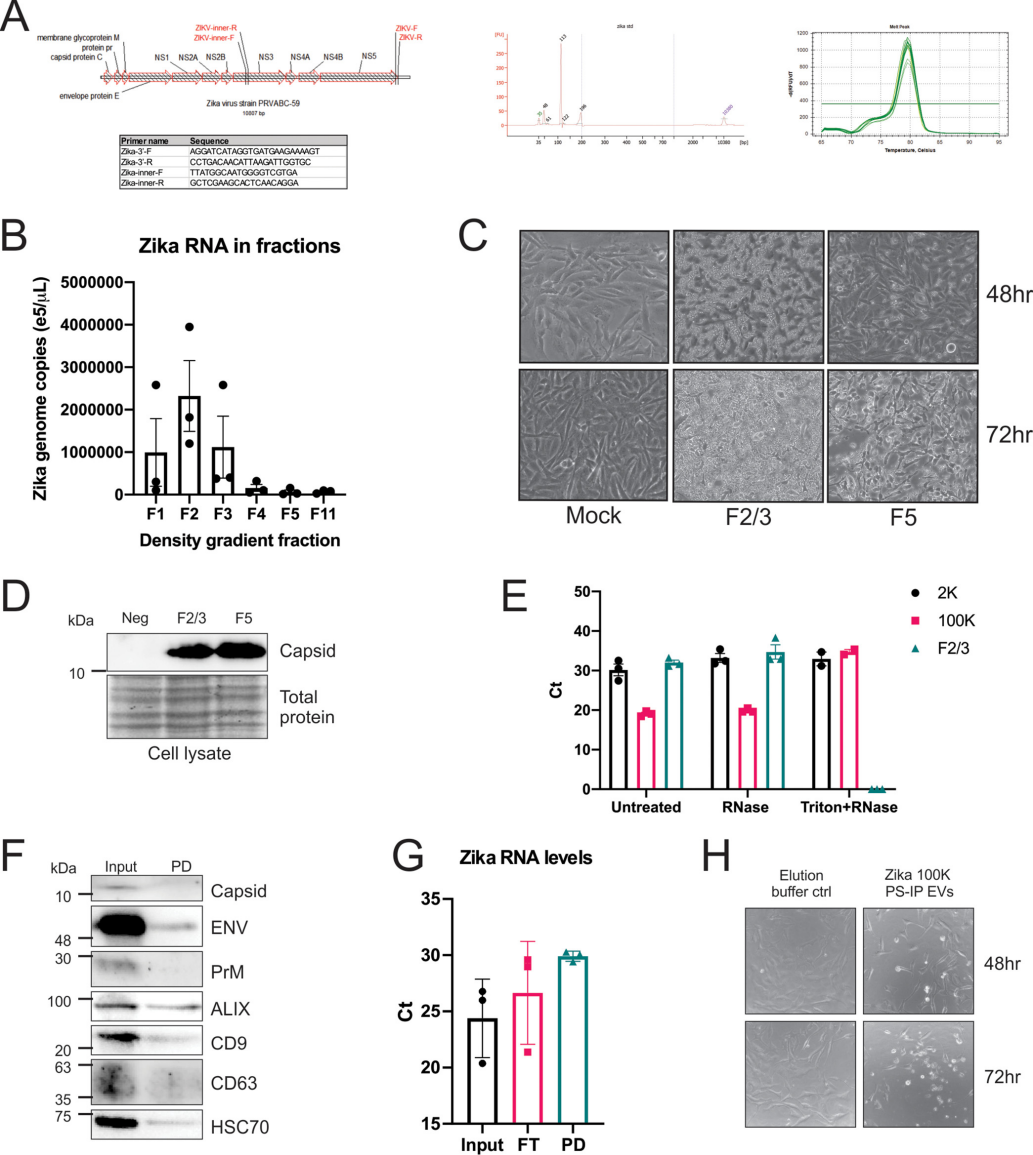
Zika virus-modified small EVs contain viral RNA and are infectious. (A) Previously published and newly designed primers were utilized for the detection of Zika virus genomes and full-length genomes. (B) Zika virus genome copy number from gradient fractions (RT-qPCR of 3 independent experiments; error bars depict standard deviation). (C) Equal volumes of gradient fractions 2/3 and 5 were applied to uninfected SNB-19 cells. Cytopathic effects (CPEs) were monitored and documented every 24 h via live cell microscopy. (D) Cells from the transfer experiment were harvested (72 h postinfection) and lysed, and proteins were separated by SDS-PAGE. Immunoblotting of cell lysates for Zika virus capsid protein was used to confirm infection. (E) RT-qPCR results of gradient fraction 2/3, 100K, and 2K untreated, treated with RNase, and treated with Triton and RNase. (F) Immunoblot of EV markers and Zika virus proteins of the input versus PS pulldowns (PD) of 100K Zika virus EVs. (G) Zika virus RT-qPCR results of 3 independent input, flowthrough (FT), and PS 100K EV pulldown (PD). (H) Equal volumes of elution buffer only or PS pulldown 100K EVs were added to Vero cells, and CPE was captured by microscopy.

Since we established that Zika virus is modifying EVs, we wanted to then assess whether these vesicles contain infectious RNA. For this, a reverse transcription quantitative real-time PCR (RT-qPCR) method published by Xu et al. in 2016 was modified marginally and utilized to quantify Zika virus RNA genome copies in the gradient fractions (Fig. 2A) (44). We used two unique sets of primers to detect the presence of viral RNA genomes and confirmed the correct size of the end products with a Bioanalyzer (Fig. 2A). Using RT-qPCR, we were able to demonstrate that all of the top 5 fractions contained Zika virus genomes, but the copy number was greatest in fraction 2 (Fig. 2B). In addition, we were able to show that Zika virus infection can be transmitted by EVs contained in this fraction by monitoring the cytopathic effects (CPEs) and immunoblotting for Zika virus capsid protein in the cells exposed to these vesicles (Fig. 2C and D). While we would expect EVs containing positive-sense Zika virus genome to be infectious regardless of how it enters a cell, we cannot exclude the possibility that some virions contained in the gradient fractions could also produce productive infection. However, since no capsid protein was detected in the fractions and EV proteins were enriched, we anticipate this to be negligible.

RNA can be found extracellularly in a non-membrane-encapsulated form, so we wanted to evaluate whether the Zika virus RNA is actually contained within the vesicles or simply associated with the outside of the membrane. In order to assess this, RNA was extracted from Zika virus fraction 2/3, 100K, and 2K EVs that were untreated, treated with RNase, or treated with Triton and RNase. The 2K EVs were obtained from the pellet following a 2,000 × *g* spin and likely include large EVs as well as capsid-containing virions. The 100K pellet is obtained following the differential centrifugation but prior to gradient purification and is expected to contain both capsid-containing virions and capsid-less Zika virus-modified EVs. Treating the EVs with RNase resulted in no significant increase in threshold cycle (*C_T_*) in any of the EV groups, whereas the addition of Triton prior to RNase treatment resulted in a lack of RNA detection in the fraction EVs (Fig. 2E). As would be expected of encapsulated genomes, there was also a detergent- and Triton-resistant genome population in the 100K fraction, indicating the presence of virions in this pellet (Fig. 2E). These results support Zika virus RNA being contained within vesicles in the fraction 2/3 EVs since the RNA was not degraded by RNase treatment unless the membrane was first solubilized with detergent. The inability to detect any RNA in the Triton-treated fraction 2/3 sample also supports that few if any genomes are present within a mature capsid found in virions.

To provide more evidence that these EVs represent a distinct infectious population, we then decided to perform an alternative isolation method utilizing beads conjugated with a phosphatidylserine (PS) binding protein to pull down EVs because EVs are known to have exposed PS on their surface (45, 46). The 100K pellet was utilized for these experiments since it is expected to contain both virions and Zika virus-modified EVs. Immunoblotting of the PS pulldown EV proteins revealed that EV markers (Alix, CD63, CD9, and HSC70) and Zika virus envelope were captured but the Zika capsid and prM proteins were detected only in the input (Fig. 2F). As a control, we also performed a coimmunoprecipitation (co-IP) of the virus stock with Zika virus Env antibody and were able to detect capsid (see Fig. S1A in the supplemental material). We then performed qPCR of these EVs and found that Zika virus RNAs were detected in both the pulldown and flowthrough, suggesting that this pellet likely contains both virions and Zika virus-modified EVs (Fig. 2G). We analyzed the infectivity of the EV pulldowns by introducing them to naive Vero cells following elution from the beads and monitoring CPE over 72 h (Fig. 2H). CPE was visualized in the Vero cells exposed to the Zika virus PS-pulled-down EVs but not in the buffer control, indicating that these EVs are indeed infectious (Fig. 2H). Altogether, these data provide evidence for Zika virus modifying EV cargo to produce infectious EV subpopulations distinct from virions.

10.1128/mSphere.00192-21.1 FIG S1 Capsid was detected in the IP of virus stock, and capsid localization was not altered in CD81 knockdown and overexpression cells. (A) IP of virus stock with Zika virus ENV and flavivirus group antigen antibody displaying the detection of capsid. (B) IF of fixed SNB-19 inducible shCD81 knockdown cells and live cell imaging of mCherry CD81 SNB-19 overexpression cells 48 h after infection with Zika virus. Download FIG S1, TIF file, 2.8 MB. Copyright © 2021 York et al. 2021 York et al. https://creativecommons.org/licenses/by/4.0/ This content is distributed under the terms of the Creative Commons Attribution 4.0 International license.

### Knockdown of tetraspanins increases Zika virus infectivity and secretion of infectious particles/vesicles.

**FIG 3:**
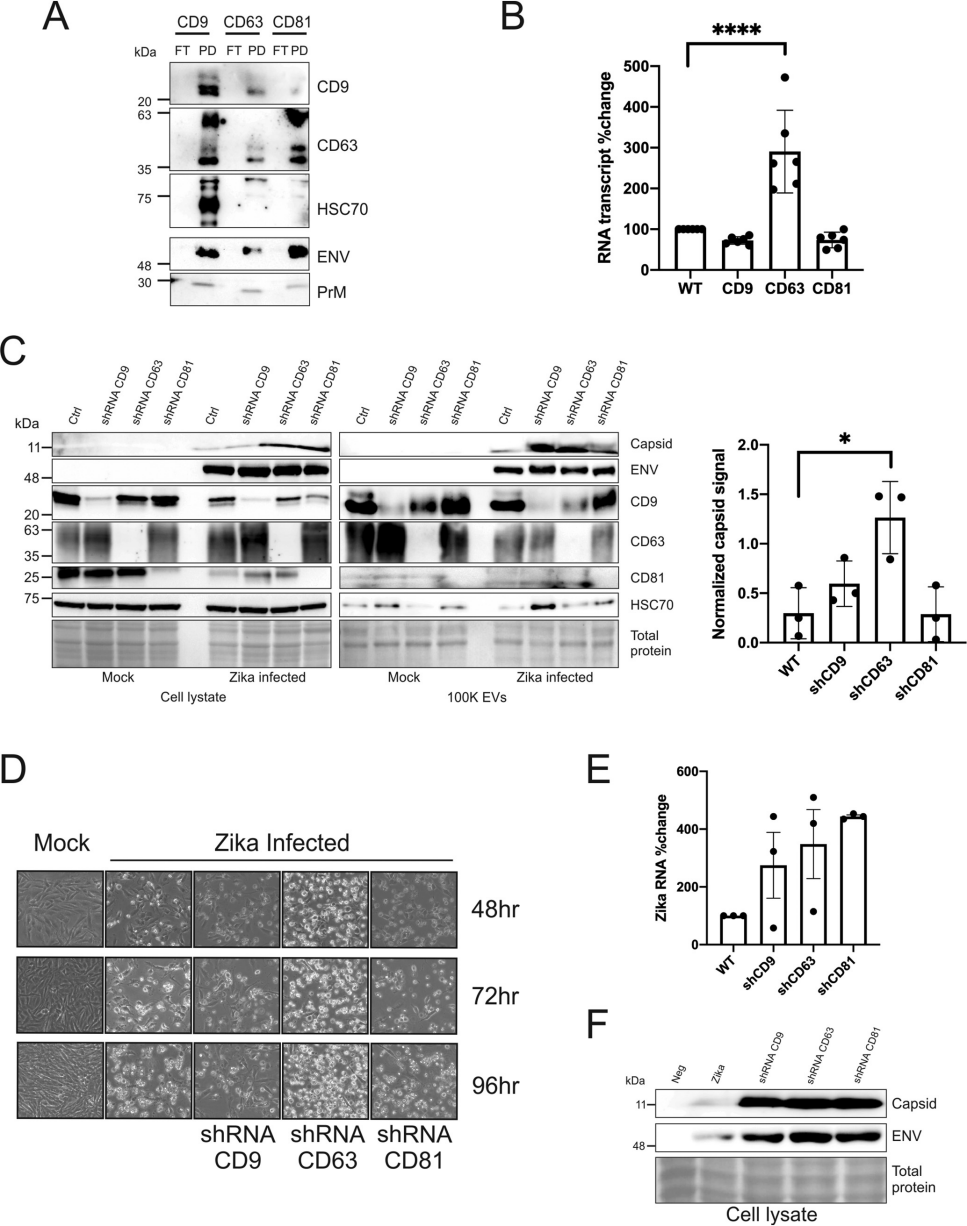
Knockdown of tetraspanins increases Zika virus infectivity and secretion of infectious particles/vesicles. (A) Immunoblot of tetraspanin CD9, CD63, and CD81 EV immunoprecipitated proteins probed for EV markers and Zika virus proteins. (B) RNA was extracted from cells 48 h after Zika virus infection, and RT-qPCR was performed to obtain transcript levels of CD9, CD63, and CD81 (error bars represent standard errors of the means [SEM] from 3 independent experiments; samples normalized to control and control set to 100); ****, *P* < 0.0001. (C) Immunoblots of cell and EV 100K lysate harvested at 72 h from uninfected or Zika virus-infected control (Ctrl) and inducible tetraspanin knockdown cells that were probed for EV markers and Zika virus proteins. Quantitation of 100K EV Zika virus capsid levels of 3 independent experiments (error bars displayed as SEM; *, *P* < 0.05). (D) Tetraspanin inducible shRNA knockdowns and control cells were monitored for CPE via live cell imaging every 24 h for 4 days after infection with Zika virus or mock. (E) Quantitation of the percent change in Zika virus 100K EV RNA levels of 3 independent experiments (error bars display SEM from 3 independent experiments; samples normalized to control and control set to 100). (F) Immunoblot of cell lysate from cells exposed to EVs from tetraspanin knockdown cells.

To further characterize the subpopulations of Zika virus-modified EVs, we then performed immunoprecipitation of the 100K pellet with beads conjugated to antibodies to CD9, CD63, and CD81 since tetraspanins are known EV markers that have significant roles in EV biogenesis. Western blotting (WB) of the IP proteins demonstrated that both EV markers (CD9, CD63, and HSC70) and Zika virus proteins (envelope and prM) were captured in all three pulldowns and not detected in the flowthrough (Fig. 3A). These data suggest that Zika virions may contain tetraspanins in their membranes since little protein was detected in the flowthrough. However, this may not be surprising since prior studies have shown that some enveloped viruses bud from tetraspanin-enriched microdomains and thus contain tetraspanins on the surface (47, 48).

EV trafficking and biogenesis proteins, including tetraspanins, have been previously shown to have a role in the packaging of viral proteins into EVs and virus assembly and egress (12, 49). As Zika virus appeared to be enhancing the production of EVs from infected cells with infectious genomes, we reasoned that the virus may be influencing expression of these genes. Therefore, we assessed whether Zika virus infection altered the transcript levels of tetraspanin genes in cells. The 48-h time point was chosen as substantial CPE usually occurs at 72 h and the levels should be evaluated while the cells are infected but before extensive cell death. The results show that CD63 transcript levels increase considerably but CD9 and CD81 levels decrease slightly (Fig. 3B). Since these transcripts were measured at 48 h during early infection, it is possible the virus requires CD63 during a stage of the replication cycle. The elevated CD63 transcripts could also represent a host reaction to the viral infection rather than an intended outcome of the virus. The reduced extracellular levels of CD63 suggest that MVBs containing CD63 may be being shuttled to the lysosome or autophagosome instead of the cell surface for release; however, this requires further investigation.

To test the importance of tetraspanins in Zika virus replication, a doxycycline-inducible short hairpin RNA (shRNA) system was employed to knock down the tetraspanin proteins CD9, CD63, and CD81 in infected cells. The tetraspanin knockdown cell lines were infected with a multiplicity of infection (MOI) of 0.5. No differences in CPE or Zika virus protein levels were detected between the infected SNB-19 wild-type (WT) and inducible shRNA scramble cells (data not shown). Immunoblot assays of the cell and EV lysate demonstrate efficient knockdowns of the tetraspanins (Fig. 3C). In addition, there were increased capsid levels in the CD9 and CD63 knockdowns, but a statistically significant increase was observed only for the EV shCD63 lysate (Fig. 3C). Although alterations in capsid protein levels were quantifiable, Zika virus envelope levels appeared relatively unaffected (Fig. 3C). The CPE was monitored by live cell imaging for 4 days, and substantial CPE was visualized in the CD63 knockdown cell line compared with the other cell lines at all three time points (Fig. 3D). RT-qPCR of the EVs revealed a considerable increase in the Zika virus RNA from all three of the tetraspanin shRNA cells (Fig. 3E). Therefore, tetraspanin, especially CD63, appear to modulate EV, capsid, and genome release from infected cells.

Next, we wanted to analyze the infectious capability of the viral particles/vesicles. This was accomplished by transferring tetraspanin knockdown total EVs to naive SNB-19 cells and harvesting the cells 96 h postexposure. Immunoblot assays of the knockdown EV-treated cells reveal dramatic increases in Zika virus capsid and envelope protein levels compared with the control (Fig. 3F). Altogether, these data suggest that EV tetraspanins CD9, CD63, and CD81 modulate Zika virus replication and spread.

### Overexpression of tetraspanins decreases Zika virus infectivity and transmission.

**FIG 4:**
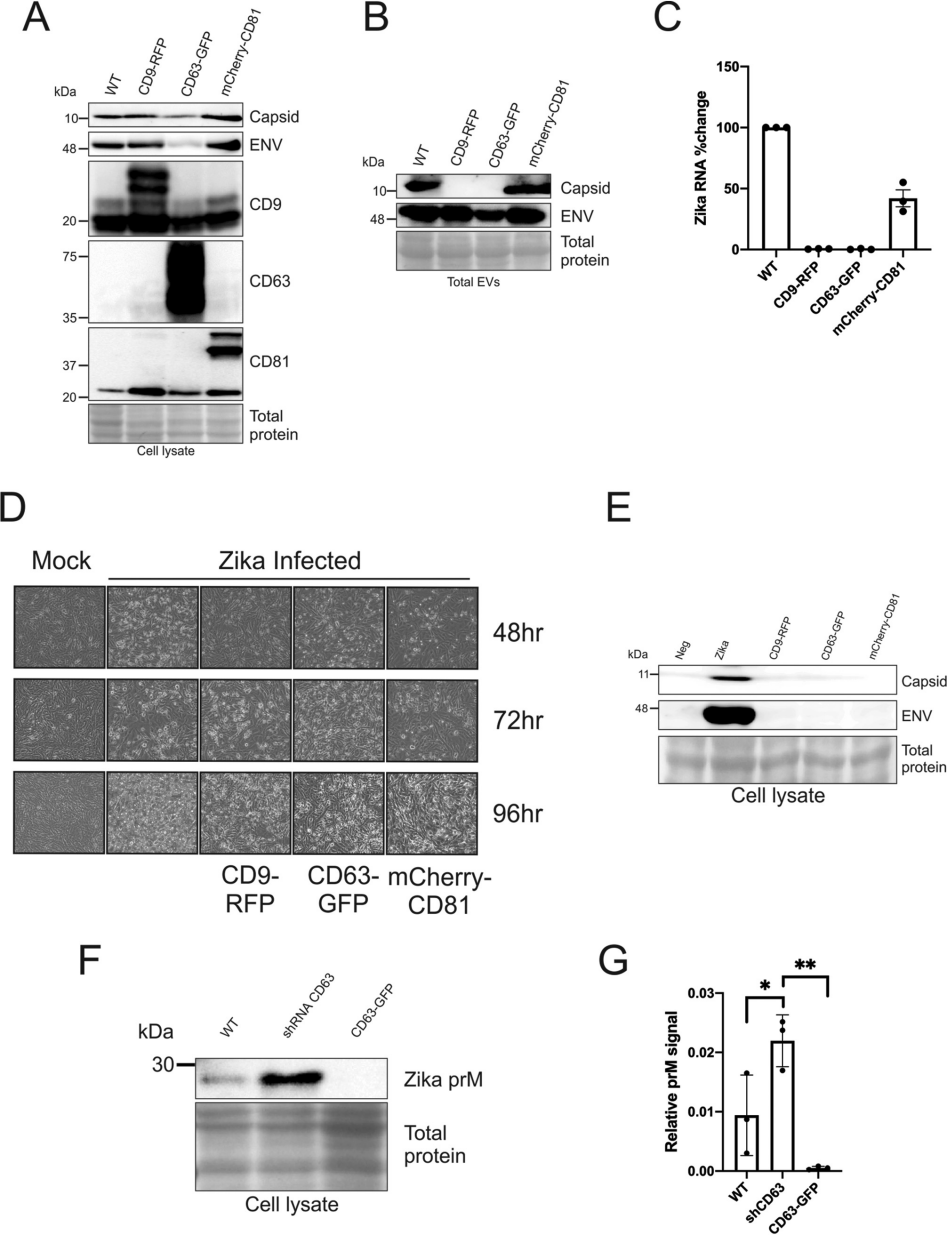
Overexpression of tetraspanin levels decreases Zika virus infectivity and transmission. (A) Immunoblots of cell lysate harvested at 72 h from uninfected or Zika virus-infected tetraspanin overexpression and control cells that were probed for tetraspanins and Zika virus proteins. (B) Immunoblot of total EV pellet from infected control and tetraspanin overexpression cells probed for Zika virus capsid and envelope (total EVs obtained from PEG precipitation of medium of uninfected/infected cells following 1,000 × *g* spin for 10 min). (C) Quantitation of the percent change in Zika virus total EV RNA levels of 3 independent experiments (error bars display SEM from 3 independent experiments; samples normalized to control and control set to 100). (D) Tetraspanin overexpression and control cells were monitored for CPE via live cell imaging every 24 h for 4 days after infection with Zika virus or mock. (E) Immunoblot of cell lysate from cells exposed to EVs from tetraspanin overexpression and control cells. (F and G) Immunoblot of Zika virus prM protein (F) and quantification of 3 independent samples of WT and CD63 knockdown and overexpression cell lysates (G). Statistical significance was determined using one-way ANOVA (*, *P* < 0.05; **, *P* < 0.01).

Since knocking down tetraspanins seems to increase EV and genome release, we then wanted to explore the effects of tetraspanin overexpression. When SNB-19 WT cells and SNB-19-green fluorescent protein (GFP) cells were infected, no difference was observed in the resulting CPE or cellular Zika virus protein levels, confirming that overexpression of GFP alone does not influence viral infection (data not shown). Zika virus-infected SNB-19 cells overexpressing CD9, CD63, and CD81 and total EV lysates (2K, 10K, and 100K vesicles and viral particles) were then harvested at 96 h postinfection and separated on SDS-PAGE gels. Cell lysate blots demonstrate an effective overexpression of tetraspanins in all three cell lines (Fig. 4A). Zika virus capsid and envelope levels detected in the cell lysates of the CD63 overexpression were decreased compared with the control and other cell lines (Fig. 4A). Immunoblotting of total EVs revealed a substantial decrease in capsid levels from the CD9 and CD63 overexpression cells while envelope protein was only mildly decreased (Fig. 4B). This was a striking finding since the total EV preparation should include Zika virions and Zika virus-modified EVs. This implies that overexpression of CD9 and CD63 disrupted the secretion of Zika virions since capsid is a critical structural component of the virion (50). The total EVs of all three overexpression cells were analyzed by RT-qPCR and found to have sizable decreases in percentage of Zika virus RNA detected compared with the control (Fig. 4C). Altogether, these results suggest that Zika virus infection and transmissive capabilities are compromised due to overexpression of CD9, CD63, and CD81.

Since tetraspanins appear to regulate vesicle, capsid, and genome release, we then wanted to assess whether knockdown alters infectivity and transmission. The overexpression cell lines were infected with an MOI of 0.5, and CPE was documented by live cell imaging for 4 days (Fig. 4D). CPE was observed but appeared to progress more slowly in all three cell lines compared with the control (Fig. 4D). We then performed the EV transfer experiments and harvested the cells 96 h postexposure. Immunoblot analysis of the lysate from the cells exposed to the overexpression tetraspanins EVs showed that all had striking decreases in Zika virus capsid and envelope proteins (Fig. 4E). These data suggest that EV tetraspanins CD9, CD63, and CD81 modulate Zika virus replication and spread.

In order to further evaluate how tetraspanins affect replication and/or egress, we compared the levels of Zika virus prM protein in the CD63 knockdown and overexpression cell lysates (Fig. 4F). We chose to focus on the effects of CD63 since this tetraspanin had the most significant impact on infectivity in the previous experiments. Zika virus prM protein is the immature form of the structural M protein of the virus particle which is cleaved upon extracellular release. Interestingly, the level of prM is significantly increased in the knockdown and decreased in the overexpression (*P* < 0.05 and *P* < 0.01, respectively) (Fig. 4F and G). These results additionally support that CD63 plays some role in Zika virus replication.

### CD63 localization is altered with Zika virus infection, and the expression levels of CD63 affect the cellular localization of Zika virus capsid protein.

**FIG 5:**
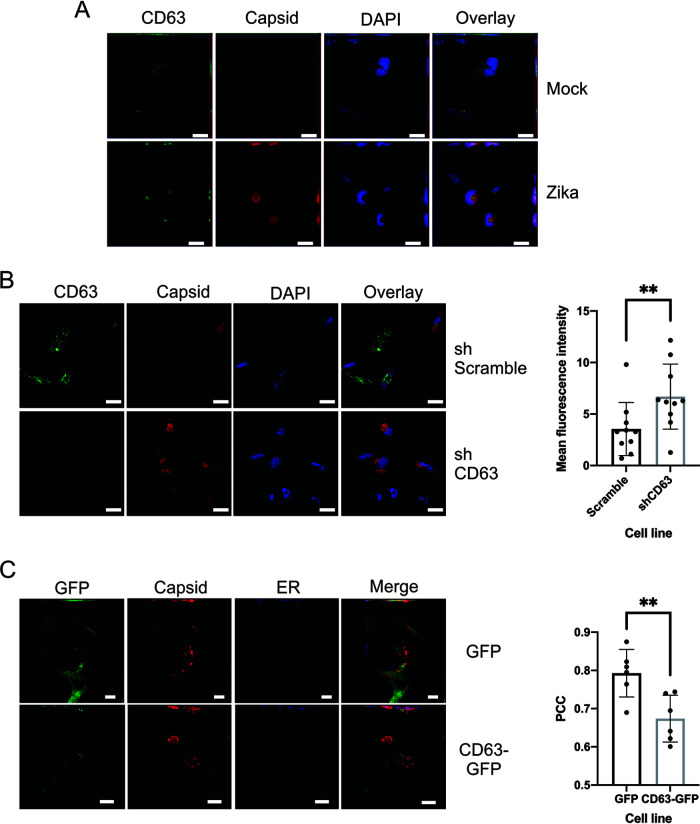
CD63 localization is altered with Zika virus infection, and the expression levels of CD63 affect the cellular localization of Zika virus capsid protein. (A) IF of fixed SNB-19 cells was used to analyze endogenous CD63 and Zika virus capsid localization in WT cells 48 h after mock infection or infection with Zika virus. (B) SNB-19 inducible shRNA scramble or CD63 knockdown cells were fixed for IF for Zika virus capsid proteins 48 h postinfection. (C) Representative images and image quantification of Zika virus capsid localization in GFP control and CD63-GFP overexpression fixed SNB-19 cells 48 h following infection with Zika virus. ER, endoplasmic reticulum. Scale bars = 20 μm.

Since various levels of CD63 appear to alter Zika virus replication and capsid release, we next examined the localization of endogenous CD63 and Zika virus capsid in the mock- and Zika virus-infected cells. Surprisingly, upon Zika virus infection, CD63 is sequestered to the core of the perinuclearly located viral replication site and surrounded by capsid protein (Fig. 5A). This was a remarkable finding since CD63 was found to be dispersed throughout the cytoplasm of the mock-infected cells (Fig. 5A). These results shed new light on the influence of CD63 on the viral replication site and virally induced cytoplasmic reorganization.

**FIG 6:**
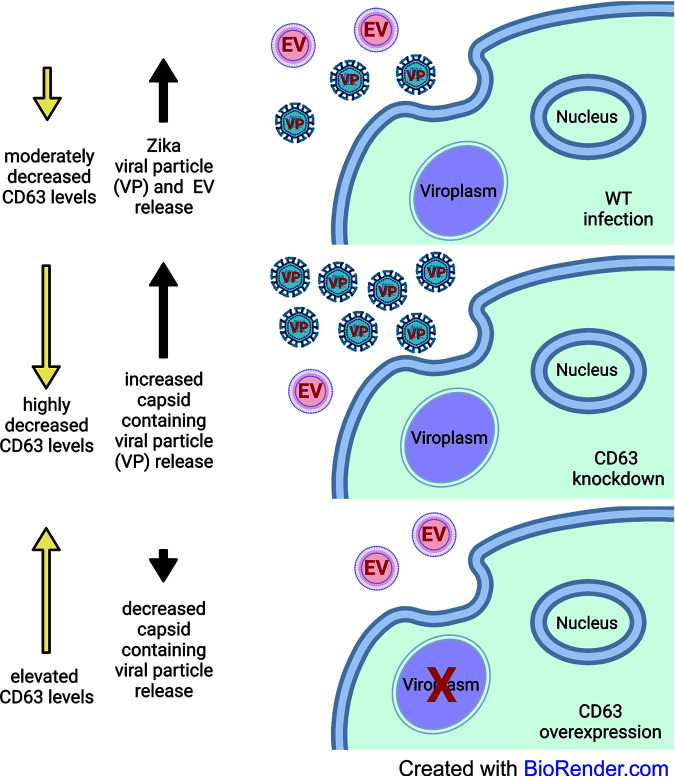
Graphical model of the influence of CD63 during Zika virus infection. Visual representation of Zika virus infection in WT and CD63 knockdown and overexpression cells and the associated effects of CD63 on viral particle/EV release. Figure created with BioRender.

To further ascertain the role of CD63 in Zika virus infection, Zika virus envelope and capsid protein localization was assessed in the SNB-19 CD63 knockdown and overexpression cells. When CD63 was knocked down, capsid was found to form tight cytoplasmic viral replication sites compared with the scramble control (Fig. 5B). However, in the CD63 overexpression cells, these sites were observed only in cells that lacked or had low CD63-GFP expression (Fig. 5C) In fact, in the cells that had high CD63 expression, capsid protein levels were found to be minimal (Fig. 5C). Yet, low levels of CD63 expression revealed a similar phenotypic localization that was observed when endogenous CD63 levels were probed in the WT cells (Fig. 5A and C). Image quantification of the correlation coefficient of GFP/capsid and GFP-CD63/capsid revealed significant decreases in CD63/capsid correlation (Fig. 5C). These data suggest that elevated levels of CD63 disrupt or prevent the formation of capsid-containing replication sites. To demonstrate that this phenomenon is not a general finding in the knockdown and overexpression of tetraspanins, we repeated the imaging in our CD81 knockdown and overexpression cells (Fig. S1B). The alterations to Zika virus capsid as seen with CD63 were not observed in the CD81 cells (Fig. S1B). Taken together, these results further support CD63 having a distinctive regulatory role in Zika virus replication and the production of virions and Zika virus-modified EVs. In order to help visualize our hypothesis of the role of CD63 in Zika virus infection, we included a model depicting the influence of CD63 in Zika virus infection in our WT, knockdown, and overexpression cells (Fig. 6).

## DISCUSSION

As the field of EVs continues to expand, there has been accruing interest in the pathological role of EVs in infectious diseases. Numerous studies have recently documented the utilization of EVs in the life cycle of various viruses (16, 17, 47, 48, 51, 52). This is not overly surprising since viruses are obligate intracellular parasites and EVs are among the normal processes of cells. Yet, much of the underlying molecular mechanisms behind how viruses utilize these pathways and for what purpose is still being investigated. We sought to delve deeper into these questions and not only characterize the modification of vesicles by Zika virus but also explore the importance of specific EV trafficking and biogenesis proteins in Zika virus infection and transmission.

Although Zika virus has been recently found to secrete infectious EVs, a detailed systematic analysis of the effects of Zika virus infection on EV subpopulations has not been performed. Zhou and colleagues released their findings that EVs harvested from Zika virus-infected cells contain Zika virus genomes and are infectious (53). Our data support these findings but expand on them by demonstrating that Zika virus infection modifies EV biogenesis and cargo. Furthermore, we show the diversity of vesicle populations released from Zika virus-infected cells, all of which could have distinct functions and influence pathogenic outcomes. Interestingly, van der Grein et al. reported that picornaviruses, which are also RNA viruses, utilized different EV biogenesis pathways for the secretion of infectious particles with distinct cargo and roles in infection (52). Hence, these discrete vesicle populations may have various roles in maintaining Zika virus infection and continuing the viral life cycle. Although alphaviruses have been previously demonstrated to release infectious microvesicles that are lacking capsid protein (54), to our knowledge this is the first report of Zika virus-infected cells releasing infectious small EVs that are capsid deficient. While we cannot completely rule out the possibility that the small light EVs purified do not contain some virions that produce active infection, virion contamination alone cannot account for the high level of genomes and infectivity found in this EV subpopulation. Other lines of evidence that support a capsid-less EV subpopulation that is infectious include (i) the inability to detect capsid in this fraction, (ii) the detection of Zika virus genomes in this fraction that were sensitive to RNase treatment following membrane disruption, (iii) the inability to detect capsid-containing virions by EM, and (iv) the ability to pull down a capsid-deficient infectious EV population with PS from the 100K pellet. Regardless of whether capsid-less EVs transmit Zika virus infection, it is clear from our work and others that Zika virus dramatically modifies EVs produced by cells, which likely contributes to Zika virus spread and pathogenesis within the host.

As mentioned, Zika virus has a complex life cycle with its reliance on vectors as its primary means of transmission. But this virus also has a broad tropism and the capacity to cross cellular barriers such as the blood-brain barrier (BBB) and placenta. It is these capabilities that have led to the severe manifestations of the disease including congenital microcephaly. In 2016, Tang et al. demonstrated that Zika virus could infect neural progenitor cells, which leads to cell cycle dysfunction and cell death (55). These results reveal how Zika virus is able to damage the developing fetal brain, but the important question of how the virus can cross the placenta and infect the fetus remains unanswered. Interestingly, studies indicate that Zika virus is unable to infect placental trophoblasts and that these cells secrete factors that support an antiviral cellular environment within the placenta (56, 57). However, EVs have been reported to cross the placental barrier (23, 24). Here, we have demonstrated that Zika virus utilizes different biogenesis pathways in cells for the production of virions and virally modified EVs. Consequently, it is possible that the Zika virus-modified EVs have different tropic capabilities.

Recent work from Zhou and colleagues revealed that neutral sphingomyelinase 2 (nSMase-2) was required for efficient viral persistence and transmission in mouse cortical neurons (53). Huang and colleagues also showed that treatment with the nSMase-2 inhibitor GW4869 negated the Zika virus-induced EV secretion and reduced the infectivity (58). These findings support EV biogenesis pathways as having a significant role in the transmission of Zika virus since ceramide, the target of nSMase-2, drives budding of vesicles into the MVB (59). Ceramide is implicated in the ESCRT-independent EV biogenesis pathway because it is believed to spontaneously produce budding events when concentrated in the membrane due to the curvature induced from the rigid cone-shaped structure of the lipid head group (59).

Although in this study we focused primarily on the tetraspanin-mediated trafficking and biogenesis pathways, it is worth noting that it will be important in future studies to examine the role of ESCRT components in Zika virus infection as tetraspanins can follow ESCRT-dependent and -independent pathways. Other studies have reported the importance of ESCRT proteins in flavivirus propagation and egress (49, 60). In the study by Thepparit et al., apoptosis-linked gene-2-interacting protein X (Alix) was discovered to be upregulated in cells infected with dengue virus (49). Dengue virus infection was also shown to be enhanced upon overexpression of Alix and restricted when Alix was knocked down in the cells (49). In addition, Tabata and colleagues found that knocking down tumor susceptibility gene 101 (TSG101) and other specific ESCRT-III proteins resulted in significant decreases in dengue virus and Japanese encephalitis virus titers (42). Therefore, it is quite plausible that there are essential functions for these ESCRT proteins in the Zika virus life cycle. It would be also insightful to explore the role of these proteins in both virion and Zika virus-modified EV secretion.

In addition to ceramide and ESCRT proteins, tetraspanins have been found to be crucial to other viruses and the secretion of virally modified EVs (12, 41). For instance, our lab previously demonstrated that CD63 is required for the efficient secretion of Epstein-Barr (EBV) viral oncoprotein latent membrane protein 1 (LMP1) in EVs (12). Tetraspanins have also been implicated in the budding and egress of many viruses including HIV, human cytomegalovirus (HCMV), influenza virus, hepatitis A virus, HSV-1, and others. Additionally, tetraspanins serve important functions in virus entry. For example, hepatitis C virus utilizes CD81 to infect hepatocytes (61). Recently, Vora and colleagues also discovered that dengue virus-infected mosquito cells release tetraspanin (Tsp29Fb)-enriched EVs that are infectious to both mosquito and human cells (62). Since the initial characterization of the Zika virus-modified EVs found alterations in tetraspanin protein levels in the EVs from the Zika virus-infected cells, it is possible that these proteins have a role in Zika virus infection and transmission.

The specific contributions of tetraspanins to Zika virus replication can be difficult to decipher due to the complexity of the roles and functions of tetraspanins in cellular processes. Tetraspanins have been shown to form protein and lipid clusters in membranes called tetraspanin-enriched microdomains (TEMs), which are thought to serve as platforms for EV cargo to congregate as well as sites for virus budding and cell entry (35, 63, 64). It is possible that excessively high or low CD9, CD63, or CD81 levels may disrupt the composition of these TEM sites, leading to changes in cargo recruitment to membrane microdomains. This may result in the accumulation of components utilized by the virus, thus allowing for more efficient virion production. Otherwise, if a large portion of cellular resources is being funneled through specific EV pathways due to the overexpression of tetraspanins, the virus may be unable to adequately recruit the materials needed for replication and virion assembly.

Alternatively, CD63 levels may have an influence on viral morphogenesis. Interestingly, there has not been a packaging signal established for the attachment of the RNA to the capsid in flaviviruses, and currently, it is thought that the capsid accumulates the RNA for compaction in the virion (65–67). It also has been proposed that surface glycoproteins actually drive morphogenesis (68, 69). Our data have demonstrated a disruption of capsid-containing particles in the presence of high levels of CD63 with little effect on extracellular envelope levels. Therefore, it is possible that CD63 may be impacting the capsid-RNA interaction while still allowing for viral morphogenesis of capsid-deficient particles. Future studies are warranted on the role of CD63 and other tetraspanins in Zika virus biogenesis.

In addition to virion assembly, it is also possible that the altered membrane composition may affect viral binding, endocytosis, or fusion during entry events. The interferon-induced transmembrane proteins (IFTMs) have been reported to accumulate in TEMs as an innate immune response (70). Therefore, it is possible that this accumulation of IFTMs in the TEMs could disrupt tetraspanin interactions and receptor recruitments, leading to the restriction of viral entry (71). Furthermore, the availability of cellular receptors or endocytic machinery may be affected by alterations in tetraspanin levels, thereby suppressing virus entry.

Our lab previously reported that CD63 has a regulatory role in autophagy and that CD63 bridges the endosomal and autophagic pathways (72). Interestingly, the study by van der Grein and colleagues found that the picornavirus light (EV) infectious population was enriched in autophagic markers and indicated that these viruses promote autophagy in cells (52). We just identified that CD63 levels in the different vesicle populations varied when the cells were infected with Zika virus, and Zika virus has also been reported to induce autophagy (73). Consequently, it is possible that Zika virus is utilizing CD63 in the autophagic secretory pathways for the release of autophagically derived infectious particles. When we compared the Zika virus genome and protein levels of the small gradient-purified EVs from the cells with the overexpression and knockdown of CD63, we found the levels to be significantly lower. These findings suggest that specific levels of CD63 may be required for the secretion of these Zika virus-modified EVs.

Interestingly, the immunofluorescence results of endogenous CD63 levels revealed that endogenous CD63 is located in the core of the virus replication unit. Notably, a study by Cortese and colleagues demonstrated that the core of the virus replication unit contains a pore where newly synthesized Zika virus positive-sense RNA is released (74). When we examined the localization of the Zika virus envelope and capsid proteins in the CD63 knockdown and overexpression cells, CD63 levels appeared to negatively correlate with capsid levels, further supporting high levels of CD63 as being disruptive to the establishment of the Zika virion replication sites. These findings support our hypothesis that CD63 levels may alter the cellular trafficking pathways and assembly sites utilized by the virus for the production and secretion of different types of infectious particles.

It appears that CD63 is a means by which Zika virus can balance the secretion of virions and infectious EVs. Having lower levels of virion release and infectious Zika virus EVs may provide better immune evasion and persistence as well as the ability to cross cellular barriers (75). Evolutionary long-term strategies tend to favor adaptability and persistence especially for viruses with complex life cycles such as arboviruses. Arboviruses require a vector to maintain the life cycle, but having other routes of transmission, i.e., vertical or sexual, increases the chance of transmission. This would be evolutionarily favorable, and Zika virus appears to be exploiting EV biogenesis pathways to increase its transmission and persistence capabilities.

Overall, this study provides a detailed analysis of the extracellular vesicles released from Zika virus-infected cells and new insights into Zika virus cell-to-cell transmission. It also provides the first evidence for the importance of host tetraspanin proteins in Zika virus replication and spread. Obtaining a better understanding of how Zika virus utilizes tetraspanins and EV pathways to transport infectious genomes and viral and host cargo is likely to offer novel targets to control infection and disease.

## MATERIALS AND METHODS

### Cell culture.

Vero E6 cells (a kind gift from Hengli Tang, Florida State University) were cultured in Dulbecco modified Eagle medium (Sigma; D5796) supplemented with 10% fetal bovine serum (FBS; Gibco; 26140-079), 2 mM l-glutamine (Corning; 25-005-CI), 100 IU of penicillin-streptomycin (Corning; 30-002-CI), and 100:0.25 μg/ml antibiotic/antimycotic (Corning; 30-002-CI). The SNB-19 cells were obtained from Charles River Laboratories, Inc., under contract of the Biological Testing Branch of the National Cancer Institute. SNB-19 cells were grown in RPMI 1640 (Sigma; R8758) supplemented with 10% fetal bovine serum (Gibco; 26140-079), 2 mM l-glutamine (Corning; 25-005-CI), 100 IU of penicillin-streptomycin (Corning; 30-002-CI), and 100:0.25 μg/ml antibiotic/antimycotic (Corning; 30-002-CI).

### Extracellular vesicle-depleted FBS.

EV-depleted FBS was used in all of the experiments in which EVs were harvested. FBS was centrifuged at 100,000 relative centrifugal force (rcf) for 20 h in a SW32Ti rotor and then filtered through a 0.2-μm filter.

### Plasmid cloning.

pLenti X1 shRNA plasmids are as follows (underlining indicates target sequence): CD9_shRNA_fwd, 5′GATCCC CAAGAAGGACGTACTCGAAAC GTGTGCTGTCC GTTTCGAGTACGTCCTTCTTG TTTTTGGAAA; CD9_shRNA_rvs, 5′AGCTTTTCCAAAAA CAAGAAGGACGTACTCGAAAC GGACAGCACAC GTTTCGAGTACGTCCTTCTTG GG; CD63 shRNA_fwd, 5′GATCCCCAACGAGAAGGCGATCCATAAGTGTGCTGTCCTTATGGATCGCCTTCTCGTTGTCTTTTTGGAAA; CD63 shRNA_rvs, 5′AGCTTTTCCAAAAACAACGAGAAGGCGATCCATAAGGACAGCACACTTATGGATCGCCTTCTCGTTGGG; CD81 shRNA_fwd, 5′GATCCCACATCCTGACTCCGTCATTTA GTGTGCTGTCCTAAATGACGGAGTCAGGATGTTTTTTGGAAA; CD81 shRNA_rvs, 5′AGCTTTTCCAAAAAACATCCTGACTCCGTCATTTAGGACAGCACTAAATGACGGAGTCAGGATGTGG.

### Retrovirus production.

Transduction retrovirus particles were collected from HEK293T cells following Lipofectamine 3000 transfection of expression plasmids (pLenti CMV TetR BLAST and pLenti X1 shRNA plasmids) and packaging plasmids pMD2.G (Addgene; number 12259; a gift from Didier Trono) and PSPAX2 (Addgene; number 12260; a gift from Didier Trono) according to the manufacturer’s instructions (Invitrogen; L3000015). The overexpression pCT-CD9-RFP (SBI; CYTO123-PA-1) and pCT-CD63-GFP (SBI; CYTO120-PA-1) plasmids were transfected in HEK293T cells with the packaging plasmids pMD2.G (Addgene; number 12259; a gift from Didier Trono), pMDLgpRRE (Addgene; number 12251; a gift from Didier Trono), and pRSVRev (Addgene; 12253; a gift from Didier Trono) according to the manufacturer’s instructions (Invitrogen; L3000015). Medium was collected and reapplied at 48, 72, and 96 h following transfection, centrifuged for 10 min at 1,000 rcf, filtered through a 0.45-μm filter, and frozen at −80°C until use.

### Generation of cell lines.

Cells stably expressing shRNA of different genes under the control of a tetracycline-inducible promoter were created by first transducing SNB-19 cells with lentivirus particles containing pLenti CMV TetR BLAST (Addgene; number 17492). The cells underwent selection with medium containing 10 μg/ml of blasticidin (InvivoGen; ant-bl-1) for 2 weeks. These cells were then transduced with pLenti X1/zeo shCD63, pLenti X1/puro shCD9, or pLenti X1/zeo shCD81. Doubly stable cells were selected with medium supplemented with blasticidin (10 μg/ml) and puromycin (2 μg/ml) or zeocin (100 μg/ml concentration) for 2 weeks. The inducible shRNA cell lines were tested for knockdown by immunoblotting of cell lysates following 24 h of doxycycline induction. SNB-19 cells were transduced with lentiviral particles containing CD9-red fluorescent protein (RFP) (SBI, CYTO123-PA-1) or CD63-GFP (SBI, CYTO120-PA-1) and selected with media containing puromycin (2 μg/ml) for 2 weeks. SNB-19 cells were transfected (Lipofectamine 3000; Invitrogen, L3000015) with the overexpression mCherry CD81 (gift from Michael Davidson; Addgene plasmid no. 55012) construct and selected with medium containing neomycin (500 μg/ml; Corning; 30-234-CI) for 2 weeks. Overexpression cell lines were confirmed by immunoblotting of whole-cell lysate and live cell imaging.

### Zika virus stock preparation.

All experiments were conducted using the Zika virus PRVABC-59 strain (kind gift from Hengli Tang, Florida State University). Viral stocks were grown in Vero E6 cells (kind gift from Hengli Tang, Florida State University), and plaque assays were used to determine virus stock titers. The protocol for producing, harvesting, and enumerating Zika virus stocks was followed as described in the work of Agbulos et al. (76).

### Zika virus infection.

Cells were counted with an automated cell counter (Cellometer Vision, software version 2.1.4.2; Nexcelom Biosciences) prior to seeding. Cells were seeded in normal growth medium for cell type with 10% FBS. For the shRNA inducible stable cells, cells were induced with 1 μg/ml doxycycline 16 to 18 h prior to infection. After 16 to 18 h, the medium was removed and cells were infected with Zika virus (MOI = 0.01 to 0.05) or mock infected (just medium) for 1 h. The flasks were rocked every 10 min. The medium was removed, the flasks were washed with phosphate-buffered saline (PBS), and growth medium was added (1 μg/ml doxycycline was reapplied to shRNA cells). After 24 h, the medium was removed and medium containing 10% vesicle-depleted FBS (1 μg/ml doxycycline was added to shRNA cells) was applied. Following 48 to 72 h, the medium and cells were harvested for further processing.

### Extracellular vesicle enrichment.

Extracellular vesicles were isolated and characterized from cell-conditioned medium following the 2018 MISEV guidelines and as previously described (72, 77, 78). Medium was collected and centrifuged serially (500 rcf for 5 min, 2,000 rcf for 10 min, and 10,000 rcf for 30 min) to remove cell debris, apoptotic bodies, and microvesicles. Supernatant was mixed 1:1 with a 16% PEG 6000 solution (16% [wt/vol] polyethylene glycol, 1 M NaCl) for a final concentration of 8% and incubated overnight at 4°C. The medium/PEG solutions were centrifuged at 3,214 × *g* for 1 h the following day in order to obtain crude EV pellets. These pellets were resuspended in phosphate-buffered saline (PBS) and ultracentrifuged at 100,000 rcf for 2 h. The EV pellets were resuspended in particle-free PBS (or sucrose buffer if they were to be further purified on a gradient) and stored at 4°C until additional analyses.

### Iodixanol density gradient EV subpopulation separation.

For further purification and subpopulation separation by flotation density gradient, EV pellets were instead resuspended in 1.5 ml of 0.25 M sucrose buffer (10 mM Tris, pH 7.4). Gradients (10 to 30%) were constructed as previously described in detail (9, 12) using OptiPrep (Sigma; D1556). EVs in the 1.5 ml buffer were mixed 1:1 with 60% iodixanol to make a final concentration of 30% and loaded on the bottom of the gradient. Then, 1.3 ml of 20% and 1.2 ml of 10% were layered on top. The gradients were centrifuged for 1 h at 50,000 rpm in an MLS 50 rotor (15-min acceleration and deacceleration for 90-min total time). The gradients were fractionated by pipetting 490 ml from the top. Ten 490-μl fractions were collected, and the gradient pellet was resuspended as the 11th fraction. Following fractionation, samples were washed in PBS and pelleted again by ultracentrifugation at 100,000 × *g* for 2 h. Pellets were resuspended in particle-free PBS.

### Immunoblot analysis.

Whole-cell lysates were harvested by trypsinizing with 0.25% trypsin for 10 min, inactivating with growth medium, and pelleting at 500 × *g* for 5 min. Cells were lysed with radioimmunoprecipitation assay (RIPA) buffer as described previously (12). EVs were lysed using a strong lysis buffer containing urea (5% SDS, 10 mM EDTA, 120 mM Tris-HCl [pH 6.8], 8 M urea, protease inhibitor cocktail; Thermo; 78438). Cell lysate protein quantification was measured by Pierce 660-nm protein assay (Invitrogen; 22662), and the EZQ protein quantitation kit (Thermo; R33200) method was used to quantify the EV lysates. Laemmli 5× sample buffer (with or without 2% β-mercaptoethanol [BME] for reducing or nonreducing conditions) was added to cell and EV lysates before SDS-PAGE separation. Equal protein of cell or EV lysate (or volume of EVs) was loaded into SDS-polyacrylamide gels. Western blot analysis was performed as described previously (12). Ponceau S stain was applied to visualize the total protein. Blots were probed using the following antibodies: Alix (Q-19), HSC70 (B-6), CD63 (TS63), CD9 (MM2/57), CD81 (B399 1.3.3.22), calnexin (H-70), Zika virus envelope, Zika virus capsid, Zika virus prM, rabbit anti-mouse IgG, rabbit anti-goat IgG, or goat anti-rabbit IgG (Fab fragment). Table 1 contains more information on the antibodies. Blots were imaged using an Image Quant LAS4000 (General Electric) and processed with ImageQuant TL v8.1.0.0 software, Adobe Photoshop CS6, and CorelDraw Graphic Suite X5.

**TABLE 1:** Antibodies

Antibody target	Manufacturer	Product no.	Dilution
Alix	Santa Cruz	SC-49268	1:1,000
HSC70	Santa Cruz	SC-7298	1:5,000
CD63 (TS63)	Abcam	ab59479	1:3,000
CD9 (MM2/57)	Millipore	CBL162	1:3,000
CD81 (B399 1.3.3.22)	GeneTex	GTX34568	1:1,000
Calnexin (H-70)	Santa Cruz	sc-11397	1:3,000
Zika virus envelope	EastCoast Bio	HM325	1:3,000
Zika virus capsid	GeneTex	GTX133317	1:3,000
Zika virus prM	GeneTex	133305	1:3,000
Anti-flavivirus group antigen	Millipore	MAB10216	5 μg antibody to 500 μg protein (IP)
Normal mouse IgG	Millipore	12-371	5 μg antibody to 500 μg protein (IP)
Rabbit anti-mouse IgG	GeneTex	26728	1:3,000
Rabbit anti-goat IgG	GeneTex	26741	1:3,000
Goat anti-rabbit IgG (Fab fragment)	GeneTex	27171	1:3,000

### Nanoparticle tracking analysis.

The Malvern NanoSight LM10 instrument was used for the nanoparticle tracking, and the videos were processed using NTA 3.1 software with the camera level of 13 and a detection threshold of 3. Samples were diluted to be in a range of 2e8 to 1.8e9 particles, and video length per replicate was 1 min.

### Electron microscopy.

Electron microscopy grids were prepared and stained as previously described (12, 79). Grids (400 hex mesh copper; Electron Microscopy Sciences [EMS]) were exposed to vesicle preparations contained in PBS for 1 h. Following the incubation, the grids were washed with PBS three times before being fixed with 2% EM-grade paraformaldehyde (EMS; EM grade) for 10 min. The grids were washed again and incubated in a 2.5% glutaraldehyde solution (EMS; EM grade) for another 10 min. Then, the grids were washed 3 times with ultrafiltered water and stained with 2% uranyl acetate (EMS). The grids were applied to 0.4% uranyl acetate-0.13% methylcellulose for 10 min and allowed to harden at room temperature for 24 h. The imaging was performed on a CM Biotwin electron microscope.

### RNA isolation and reverse transcription.

Total RNA of cell or fraction samples was isolated by TRIzol reagent (Invitrogen) and quantified with a NanoDrop spectrophotometer. Less than 1 μg of total RNA was used for reverse transcription by qScript cDNA SuperMix (Quantabio; 95048).

### Quantitative real-time PCR and data analysis.

RT-qPCR was performed following the protocol from Xu et al. with a small modification (44). A standard 3-step cycle protocol (40 cycles of 95°C for 5 s, 60°C for 10 s, and 72°C for 20 s) was used instead of one-step fast 2-step cycles as the input was cDNA, not RNA. Another set of primers targeting the inner segment of Zika virus RNA was designed to confirm the whole genome packed in fractions. PerfeCTa SYBR green FastMix (Quantabio; 95072), assay primers, and cDNA were prepared in a 20-μl reaction mixture and run on a CFX96 qPCR machine (Bio-Rad). Zika virus RNA levels were measured by RT-qPCR and either normalized to glyceraldehyde-3-phosphate dehydrogenase (GAPDH) or calculated to copy number from an external standard with the ΔΔ*C_T_* method. A Zika virus standard curve was established to determine genome copies, and the Bioanalyzer Agilent 2100 was utilized to confirm correct end product size. PCR primer sequences are shown in Table 2.

**TABLE 2:** Primer sequences^*a*^

Name	Sequence
GAPDH-F	GGAGCGAGATCCCTCCAAAAT
GAPDH-R	GGCTGTTGTCATACTTCTCATGG
Zika-3′-F	AGGATCATAGGTGATGAAGAAAAGT
Zika-3′-R	CCTGACAACATTAAGATTGGTGC
Zika-inner-F	TTATGGCAATGGGGTCGTGA
Zika-inner-R	GCTCGAAGCACTCAACAGGA
CD63-F	CAGTGGTCATCATCGCAGTG
CD63-R	ATCGAAGCAGTGTGGTTGTTT
CD9-F	TTCCTCTTGGTGATATTCGCCA
CD9-R	AGTTCAACGCATAGTGGATGG
CD81-F	TTCCACGAGACGCTTGACTG
CD81-R	CCCGAGGGACACAAATTGTTC

a Tetraspanin primers are from PrimerBank (80).

### Triton EV experiment.

EVs were treated with RNase A (1 mU) either with or without Triton X-100 (1%) for 15 min. After adding RNase inhibitor, total RNAs were isolated by TRIzol and Zika virus RNA was quantified by qPCR as described above.

### EV immunoprecipitation.

Extracellular vesicles were harvested from the 100K pellet from Zika virus-infected SNB-19 cells as previously described. Preconjugated anti-CD9 (Invitrogen no. 10614D), CD63 (Invitrogen no. 10606D), and CD81 (Invitrogen no. 10616D) antibodies were employed to pull down EVs from the 100K Zika virus-infected pellet. The MagCapture exosome isolation kit PS (Fujifilm no. 293-77601) was utilized for the PS IP experiments. All IP experiments were performed as recommended by the manufacturer. A co-IP of the Vero stock was performed by incubating Zika virus Env (EastCoast Bio; HM325), anti-flavivirus group antigen (Millipore; MAB10216), or mouse IgG (Millipore normal mouse IgG no. 12-371) antibody with the Vero stock at a ratio of 500 μg protein to 5 μg antibody overnight at 4°C with gentle rocking. Prewashed beads were added to the incubated sample and rotated at room temperature for an hour. Following the removal of the flowthrough, the beads were washed 3 times with PBS-T and resuspended in a strong lysis buffer (5% SDS, 10 mM EDTA, 8 mM urea, 120 mM Tris HCl [pH 6.8], 3% β-mercaptoethanol). Isolated EVs were further processed by protein quantitation (EZQ kit; Invitrogen, Carlsbad, CA, USA; no. R33200), Western blotting (WB), qPCR, and infectivity as previously described.

### EV transfer experiments.

Total EVs were obtained by harvesting medium from Zika virus-infected SNB-19 (control, induced shRNA, and overexpression) cells or mock-infected cells 72 h postinfection. The medium was centrifuged at 1,000 rcf for 10 min and filtered through a 0.45-μm filter. Then, the medium was mixed 1:1 with 16% PEG and stored at 4°C overnight. The next day, the medium was centrifuged at 3,214 rcf for 1 h and total EVs were resuspended in sterile PBS.

EVs harvested from density gradients or total EVs were applied to SNB-19 cells seeded in 6-well plates. The cells were monitored every 24 h for 72 to 96 h, and images were recorded using live cell imaging on the Keyence BZ-X700/BZ-X710 microscope. Cells were harvested and lysed, as previously described, 72 to 96 h postinfection. Cell lysate protein was quantitated before being separated on an SDS-PAGE gel and immunoblotted for Zika virus proteins.

### Statistical methods and analysis software.

The Student two-sample *t* test or one-way analysis of variance (ANOVA) was used to determine significance. Prism8, Microsoft Excel, Adobe Photoshop CS6, BioRender, and CorelDraw X5 were used in the design of the figures.

### Preparation of fixed cells for confocal microscopy. (i) Infection.

SNB-19 WT, inducible shRNA, and overexpression cells were seeded on glass coverslips the day prior to infection with Zika virus. The following morning, shRNA cells were induced with 1 μg/ml doxycycline, and 4 to 6 h later, all cell lines were infected with Zika virus at an MOI of 2. Following 1-h viral incubation at 37°C, medium was replaced and doxycycline was reapplied to the shRNA cells. Coverslips were processed for immunofluorescence (IF), and live cells were imaged 48 h postinfection.

### (ii) Immunofluorescence (IF).

Medium was removed from wells containing the coverslips, and the cells were fixed with 4% paraformaldehyde for 10 min. The coverslips were washed with PBS following fixation and permeabilized for 30 min with 0.2% Triton X-100–PBS (PBS-T). Cells were blocked in 5% goat serum in PBS-T for 30 to 60 min at room temperature. Primary antibody was added to coverslips (envelope, 1:500; capsid, 1:200; CD63, 1:100 diluted in 5% goat serum/0.2% Tween-PBS) and incubated at 4°C overnight. Primary antibody was removed, and the coverslips were washed with PBS-T. Secondary anti-mouse (594, Thermo 35511; 488, Thermo 35503; 405, Rockland 610-146-002-0.5) or anti-rabbit (488, ab150077) antibody at 1:1,000 in 5% goat serum/PBS-T was applied and incubated at room temperature for 1 h. The coverslips were washed again with PBS-T and mounted or stained with 4′,6-diamidino-2-phenylindole (DAPI) (Thermo; 6648) diluted 1:10,000 in PBS for 10 min. Coverslips were applied to mounting medium (4% propyl gallate, 90% glycerol, PBS) on glass slides and allowed to harden overnight. Slides were imaged with a Zeiss LSM 880 microscope, and images were processed using Zen 2.1 Black software.
