# Supplementary figures and images for: Zika Virus Hijacks Extracellular Vesicle Tetraspanin Pathways for Cell-to-Cell Transmission
Source: mSphere. 2021 Jun 30;6(3):e00192-21. doi: 10.1128/mSphere.00192-21 (PMC8265634; doi:10.1128/mSphere.00192-21)

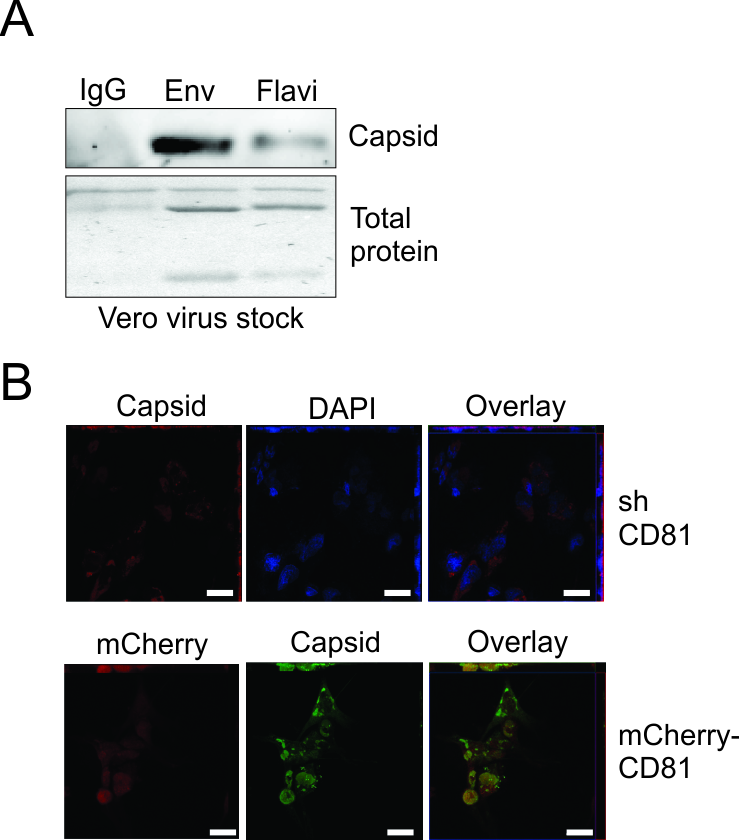

Supplement: FIG S1 [file msphere.00192-21-sf001.tif]
